# Creation and Validation of a Tool for Evaluating Caregiver Burnout Syndrome in Owners of Dogs (*Canis lupus familiaris*) Diagnosed with Behavior Disorders

**DOI:** 10.3390/ani12091185

**Published:** 2022-05-05

**Authors:** Carmen Luz Barrios, Vanessa Gornall, Carlos Bustos-López, Rosa Cirac, Paula Calvo

**Affiliations:** 1 Escuela de Medicina Veterinaria, Facultad de Ciencias, Ingeniería y Tecnología, Universidad Mayor, Camino La Pirámide 5750, Huechuraba 8580745, Región Metropolitana, Chile; vgornall@veterinaria.uchile.cl; 2 Departamento de Ciencias Básicas, Facultad de Ciencias, Universidad Santo Tomás, Av. Ejército Libertador 146, Santiago 8320000, Región Metropolitana, Chile; cbustoslopez@santotomas.cl; 3 Investigador Independiente, Sant Andreu del Palomar, 08030 Barcelona, Spain; rcirac2010@gmail.com; 4 Cátedra Animales y Sociedad, Facultad de Ciencias de la Salud, Universidad Rey Juan Carlos, 28933 Madrid, Spain; paula.calvo@antrozoologia.com

**Keywords:** caregiver burnout, behavioral disorders, human–animal bond, instrument validation, psychometric scales

## Abstract

**Simple Summary:**

Globally, over half of the human population has at least one companion animal, with the domestic dog (*Canis lupus familiaris*) standing out above other species. While this bond has mutual benefits, research shows that it can break due to canine behavioral disorders, leading to consequences including abandonment and/or euthanasia. The wear and tear implied by the physical, psychological and emotional demands when facing the care of a sick animal can lead to a continuous and prolonged level of stress, which in human medicine is referred to as caregiver burnout syndrome. Parallels can be drawn with dog owners handling animals with behavioral disorders, which makes it necessary to have a validated measurement instrument for this problem. The exhaustion of the caregiver of dependent people is evaluated through the Zarit Scale. The present study, through the Delphi method technique, modified and validated this scale to measure this overload in people with dogs with behavioral disorders. Three levels of overload were obtained (Low, Medium-Low and Medium-High Overload). Having an instrument that allows assessing the level of exhaustion of caregivers of dogs with behavior problems will provide information to help these people, and consequently their dogs, avoid the negative consequences of bond degradation.

**Abstract:**

Currently, domestic dogs (*Canis lupus familiaris*) are the most common species among companion animals. The close bond that can grow between owners and their dogs could be worn out and finally broken due to various causes. One main cause is canine behavioral problems, leading to dogs being abandoned or euthanized due to the costs faced by the owner when caring for the animal. Tools have been developed to evaluate the mental and emotional cost of caring for humans, but there is currently no validated tool for evaluating this particular problem. The objective of this study was to develop a questionnaire to evaluate caregiver burnout syndrome for owners of dogs with behavioral disorders. The methodology used consisted of drafting the tool, peer validation using the Delphi methodology and internal validation via Cronbach’s alpha. Non-linear snowball sampling was used (*n* = 156 participants). A questionnaire with 35 questions was obtained which referred to various aspects of caregivers’ lives. Regarding the description of the sample used, 50% had Low Burnout, 41% had Medium-Low Burnout and 9% had Medium-High Burnout. Furthermore, regarding the internal validation of the questionnaire, the general Cronbach’s alpha coefficient was α = 0.9468. We can thus conclude that the questionnaire is valid for measuring caregiver burnout syndrome in owners of dogs with behavioral disorders.

## 1. Introduction

Worldwide, over half the human population has at least one animal companion in their care [1]. Latin America is a world leader in pet owner percentages, with people mainly preferring the domestic dog (*Canis lupus familiaris*) as a companion [1]. For example, in Chile, 52% of homes have a dog as a companion animal [2]. It is conjectured that the canine–human bond is stronger than that with other animals, due to the years of interaction, which due to various motives (competition, cooperation, co-evolution) have generated a high level of codependence, attachment and ease of interspecies communication, to the point that they have established a niche in our society like no other [3,4,5]. It should be mentioned that, within the definitions of a bond, there is the kind that is indicated as an affectionate and enduring interaction with a unique individual, non-interchangeable with another and established according to four principles: security, intimacy, affinity and constancy [5]. In turn, a companion animal, according to the concept defined in the study by [4], is one that is “under human control, linked to a home, sharing intimacy and proximity with its caregivers and receiving affection, care and attention, guaranteeing its health”, i.e., one that becomes part of a family [4]. Studies indicated that animal caregivers perform emotional and financial efforts to enrich the human–animal bond, driven by the various benefits it provides: physiologically, psychologically, socially and therapeutically [4,6,7].

In spite of these points, research has shown that the human–dog link can be broken due to behavioral disorders in the latter party, which can lead to negative consequences for dogs, such as abandonment or even euthanasia [6,8,9,10,11,12,13]. A behavior disorder is a behavior pattern that is dangerous or annoying for humans, creating a communication dysfunction between both species and compromising their mutual well-being [13]. Authors including [11], among others, describe how some of the reasons for dogs being abandoned in shelters include behaviors considered dangerous or annoying including aggression and biting, although they can also include separation anxiety as a frequent cause [9,11]. Studies in the USA and UK show that over 80% of companion animal owners have identified some type of behavioral alteration in them, with aggressiveness standing out [8]. This suggests the importance of investigating the topic to avoid animal abandonment and euthanasia. Behavioral disorders, as well as diseases in animals, also carry physical, psychological and emotional demands on their caregivers [6,7,14,15], due to the burnout involved with informal care. Informal care is when people provide altruistic free care and attention to dependent individuals, motivated by ties of affection [16,17]. The consequence is caregiver burnout syndrome; continuous, prolonged stress caused by care for a dependent individual, causing physical, psychological and emotional exhaustion along with a rupture in the bond between the two parties [6,16,17,18,19]. It is important to consider that the concept mentioned above differs from compassion fatigue, which is understood as the ability of caregivers of other living beings such as non-human animals to notice the pain of the individual they care for [20]. The latter is very common in animal shelter staff, as a result of the link between the keepers and the animals that are housed in this type of facility. These animals frequently arrive in poor physical condition, have been subjected to previous mistreatment or finally, as a result of various reasons, they must be euthanized, which emotionally ends the people who live with them daily [21].

It should be mentioned that a review of studies on caregiver burnout syndrome in human–animal relations has produced studies by [6,14,15], which principally refer to cases of animals with chronic illnesses rather than any particular behavioral disorders, thus leaving room for interest in developing this topic [6,14,15]. Thus, considering that a dog with behavioral disorders and a person with caregiver burnout syndrome can lead to broken bonds and severe negative consequences, mainly for the animal, this study aimed to develop and validate a specific tool for the topic in question.

In a complementary way, the experience of guardians of companion animals with behavioral problems has been studied qualitatively (Buller and Ballantyne, 2020) [22]. Here, it is evident how difficult it is to be able to care for an animal with these characteristics, which can affect both the psychological and physical wear and tear of the caregiver.

Based on the information gathered, one cause that can be inferred for breaches in human–dog bonds is prolonged care for dogs with behavior disorders by overburdened owners, since people generate bonds with dogs similar to how they would with other people. This makes it possible to consider the existence of caregiver burnout syndrome for cases of human–dog bonds involving behavioral disorders. It should be mentioned that this syndrome in human bonds is highly prevalent and severe [16]. A validated measurement instrument makes it possible to confirm the existence of this syndrome in owners of dogs that have behavioral disorders. For this, the specific objectives were to develop the evaluation tool and then to validate it.

## 2. Materials and Methods

### 2.1. Participants

An in-person self-applied survey was done online (Google Forms^®^) using a Likert scale. The inclusion criteria were people over 18 years old who owned dogs diagnosed with some type of behavioral disorder in the previous six months. Exclusion criteria were people over 18 who had psychiatric and/or physical pathologies and/or were in the care of dependent people, whether this was for disease, old age and/or special care.

Non-linear snowball sampling was used to recruit the sample for this study (*n* = 156). This type of sampling consists of recruiting study subjects based on contacts of the first participants, who manage the incorporation of other people to contribute to the study. This process, which can be repeated over and over, gives the possibility for the researchers to find people that they would not otherwise have had access [23].

The participants were contacted by ethologists, who had previously cared for their animals and also recruited by acquaintances of said guardians.

This study was approved by the Scientific Ethics Committee of the Universidad Mayor de Chile on 20 March 2019 with folio 0098.

### 2.2. Methodology

Three work phases were considered: Question design, Question validation via the Delphi Method and Internal validation of the questionnaire via the Cronbach’s Alpha Coefficient. These are described herein:

#### 2.2.1. Question Modification (Step I)

The original version Zarit instrument was modified [24]. This evaluated caregiver syndrome among humans and was mainly altered regarding the species of the subject being cared for (changed from humans to dogs with behavioral disorders) and with the contextualization of each question. New questions were also made based on available scientific literature for animals [14], along with expertise from each researcher. This questionnaire classified the questions into 7 pillars: Perceived overload, abandonment of self-care (health and image), discomfort with the presence or behavior of the dog, irritability, fear for the dog’s health or future, loss of family and socioeconomic role, and guilt for not doing enough (Table 1) and consisted of 35 questions, each with five response alternatives (Likert style) (Table 2).

#### 2.2.2. Question Validation via the Delphi Method (Step II)

Following modification and the creation of new questions, the instrument was validated with a panel of experts (seven specialized professionals; three in clinical psychology and four in clinical ethology), whose observations were gathered and analyzed via the Delphi method [25]. This process began with the analysis of the Curriculum Vitae of each professional invited to participate in the study as an expert. For this, the years of experience performing in their area of expertise, current employment and professional recognition by their peers were considered. They each received an invitation letter by email detailing the different aspects of their anonymous and confidential participation, along with a brief introduction to the topic and the instructions to provide the corresponding observations. Expert feedback was done via email on two occasions, which were done with three weeks’ separation between them. The researcher group finally made a joint decision on the final structure for the tool via a qualitative concordance analysis. In other words, answer frequencies were analyzed to categorize and eventually correct the questionnaire items. Experts’ replies were categorized as answers with observations regarding Form (F), according to question format, Content (C) referring to modifications which had to do with the outlook of each specialist consulted, AQ (Add Question) if they wanted to add any questions relevant to the study and E (Eliminate) if questions were considered not germane to the survey.

#### 2.2.3. Tool Application (Step III)

Dog owners were selected who fulfilled the inclusion criteria (*n* = 156) detailed in the Participants item. Each one of them received the questionnaire via Google Forms^®^, to be answered in a self-explanatory and anonymous fashion within 45 min, following acceptance of informed consent. Subsequently, all data was gathered in Excel for statistical analysis.

### 2.3. Statistical Analysis

Descriptive statistics were applied through the construction of frequency tables and determination of percentages for the responses of each variable. The internal statistical stability of the tool was evaluated using Cronbach’s Alpha coefficient. Minitab^®^ software was used both to perform descriptive statistics and for Cronbach’s Alpha analysis. A statistical significance level of 5% was considered.

## 3. Results

### 3.1. Question Validation with the Delphi Method (Step I and II)

Regarding validation via the Delphi Method, in the first evaluation, out of a total of 35 questions, 28.57% (10 questions) were observed. In turn, 60% of the questions observed were from the point of view of Content and 30% were from Form (Table 3). After the second expert evaluation, full approval for the survey were obtained from four professionals (57%). The other 43% made new observations regarding six questions from a total of 35 (17.14%), principally about Content (50%) (Table 4).

As previously said, the criteria to keep, correct or cut questions were managed via analysis and discussion among the research team. In the first analysis round, the team decided to take the form and content observations, without adding or eliminating any questions. In the second analysis round, only the form observations were taken into account. Thus, the final questionnaire had a total of 35 questions (Table 2).

### 3.2. Internal Validation of the Questionnaire via the Cronbach’s Alpha Coefficient (Step III)

Regarding the internal validity of the evaluation tool, the general Cronbach’s alpha coefficient were α = 0.9468 while the Cronbach’s alpha coefficient per item also presented results above α = 0.9 (Table 5).

In total, 156 people were consulted in the study and classified in different levels of burnout (Low Burnout 35–69 points; Medium Low burnout 70–104 points; Medium High Burnout 105–139 points and High Burnout 140–175 points). Overall, 50% presented Low Burnout, 41% showed Medium Low Burnout and 9% had Medium High Burnout. The median was a burnout score of 73.16, which is within the range of Medium Low burnout.

## 4. Discussion

There is still no consensus regarding the construction, adaptation and validation of psychometric studies in humans [26], so these results have to be taken as part of a preliminary study, waiting for its reproducibility in latter applications to allow it to be perfected.

On the other hand, developing measurement tools for problems related with human and animal health must have the opinion of area experts—the more the better. This is why using the Delphi methodology and its collective intelligence principle is highly useful for generating consensus about these opinions regarding question formulation for a psychometric measurement tool as in our case, where both clinical ethologists and psychologists take part. However, while Delphi is a frequently used and recommended method, it is still more intuitive than rational by nature, which carries undeniable biases, principally due to varying interpretations of each question apart from those referring to their formulation and method of application [25]. It should also be noted that the Delphi method has another characteristic complicating its application, consisting in the time that experts must have to be able to repeatedly analyze the questions from the instrument being developed which can be generally scarce among these professionals.

The Cronbach’s alpha coefficient value of the present study (α = 0.9468) is considered satisfactory for both the items level and the instrument reliability level, according to the literature [6,14,24]. The usual preference is for values between 0.8 and 0.9. However, according to Luján and Cardona (2015), values above α = 0.9 indicate that there could be duplication in the questions [26]. Thus, the variation of this coefficient should be analyzed with more information according to question modification, or else redundant items should be eliminated. While questions considered redundant were eliminated, eventually this pattern may still be present due to how individual perception of wear and tear in the face of this problem carries a common emotional burden for most of the questions, however diverse they may be. This, along with other individual factors, may influence the answers received.

Finally, it is worth highlighting the importance of having tools to evaluate these items, which are validated like the ones developed in the present study. These can be useful for identifying factors which can, for example, degrade the human–animal bond in order to prevent the consequences arising from it, or else to manage treatment from both a human and an animal perspective.

The main limitation of the present study refers to the bias of incorporating participants with a low burnout level, who could be more inclined and interested in participating in a research process of this type and justify the high percentage of participants with this result. This could be due to the convenience sampling used in this study. However, it should be mentioned that this bias had no direct influence on validating the previously developed instrument, but rather would affect the burnout levels recorded by participants.

Additional and no less important limitations correspond to those difficulties already mentioned above, such as the variety of factors that affect preliminary studies in mental-emotional health, individual burnout perception, intuitive methodologies, and specific conditions demonstrated. Finally, do not forget those limitations due to the resources associated with the feasibility of carrying out this research.

## 5. Conclusions

According to the results obtained, the questionnaire developed in the present study is a valid measurement instrument to evaluate caregiver burnout syndrome among owners of dogs with behavior disorders. This is fundamental, since before now, there were no instruments with these characteristics to address a problem as notable as burnout among owners in these cases, which can directly impact the wellbeing of both the people caring for the animal and the dogs themselves.

## Figures and Tables

**Table 1:** Question classification by Tool evaluation pillar.

Name of Question Distribution Pillar	Distribution Pillar Detail	# of Questions Per Pillar
Abandonment of self-care	This pillar evaluates attrition in dog owners’ care for their image and health, relating the time that they have to dedicate to their dog.	1, 8, 12, 20, 23, 25, 28, 29, 31, 34
Perception of wear and tear	This pillar evaluates how the owner perceives stress, tiredness, angst and the resulting desire to leave the care of their dog to another person and/or stop caring for the animal.	2, 7, 14, 18, 21, 30, 32, 35
Discomfort over dog presence or behavior	This pillar evaluates when the owner feels permanently on edge and avoids exposure to any third party along with their dog.	3, 10, 24
Irritability	This evaluates how worn out the owner feels based on how much they feel bothered in contexts where they care for their animal.	4
Loss of family and social role	This evaluates owners’ wear and tear based on they perceive negative changes in their daily routines due to caring for their dog, especially decay in their social bonds, tendency to feel alone, trapped, isolated and unsupported.	5, 9, 13, 19, 27
Fear for the health or future of the dog	These questions evaluate owners’ wear and tear regarding how they perceive their own worry about the well-being of their dog.	6, 15
Economic	Evaluation of owners’ attrition based on their perception of expenses incurred by caring for the dog.	11
Guilt over not doing enough	These questions focus on evaluating caregivers’ wear and tear based on how they perceive nonconformity and non-fulfillment of their expectations as a caregiver and the lack of professional support to advise them.	16, 17, 22, 26, 33

**Table 2:** Measurement instrument for caregiver burnout syndrome among owners of dogs with behavioral disorders.

		Never	Almost Never	Sometime	Almost Always	Always
#	Question	1	2	3	4	5
1	Do you feel that because of the time you spend on your dog you no longer have enough time for yourself?					
2	Do you feel exhausted when you have to look after your dog and also handle other responsibilities?					
3	Do you feel uncomfortable with how your dog behaves?					
4	Do you feel angry when you think about your dog and everything involved in its care?					
5	Do you think that having to care for your dog negatively impacts your relationship with friends and other members of your family, even your other pets?					
6	Are you afraid for the future awaiting your dog?					
7	Do you feel exhausted when you have to be with your dog?					
8	Do you feel like your health has deteriorated due to caring for your dog?					
9	Do you believe your social life has been affected by having to care for your dog?					
10	Do you feel uncomfortable inviting friends to your house because of your dog?					
11	Do you feel like you lack enough money to care for your dog apart from your other expenses?					
12	Do you feel demotivated since your dog started to show undesired behavior?					
13	Do you feel a loss of control over your life since your dog started to show undesired behavior?					
14	Would you like to have other people take care of your dog?					
15	Do you feel insecure about how to handle your dog’s behavior?					
16	Do you feel like you should be doing more than you currently do for your dog?					
17	Do you believe you could care for your dog better than you currently do?					
18	Do you generally feel very overburdened due to caring for your dog?					
19	Do you feel unsupported or lonely because you have to care for your dog?					
20	Do you feel that due to the time you spend on your dog you no longer have enough time to care for your physical appearance?					
21	Do you often consider giving your dog up for adoption?					
22	Do you feel like you’ve lacked professional support from veterinarians or other similar professionals to face this situation?					
23	Since your dog began showing unwanted behavioral problems, have you had trouble sleeping?					
24	Do you often feel like you have to remain alert and vigilant to avoid any incidents caused by your dog’s behavior?					
25	Do you feel tired from caring for your dog?					
26	Do you feel responsible for your dog’s behavior problems?					
27	Do you believe you modify your lifestyle by caring for your dog?					
28	Do you believe your quality of life has declined due to caring for your dog?					
29	Do you feel stressed or nervous when facing your dog’s care?					
30	Do you often avoid interacting with your dog?					
31	Do you feel anxiety when you think about having to go home and care for your dog?					
32	Do you feel like you won’t be able to take care of your dog for much longer?					
33	Do you feel incompetent to care for your dog?					
34	Do you feel distraction or lack of concentration in other activities since your dog began to have behavior problems?					
35	Do you have recurring desires to get rid of your dog?					
Total Score						
Level Obtained						

**Table 3:** Observation frequencies by category (%). First round of observations.

	# of Questions (Item 2)									
Observation Type	10	12	14	15	21	22	25	32	33	35
Form	14.28%	28.57%		14.28%						
Content	14.28%		14.28%				14.28%	14.28%	14.28%	14.28%
Add question						14.28%				
Eliminate question					14.28%					

Total of experts 7 (100%).

**Table 4:** Observation frequency by category (%). Second round of observations.

	# of Questions (Item 2)					
Observation Type	4	19	21	22	25	35
Form	14.28%				14.28%	
Content			14.28%	14.28%		14.28%
Add question		14.28%				
Eliminate question						

Total of experts 7 (100%).

**Table 5:** Cronbach’s alpha values obtained by questions.

Questions (#)	Cronbach’s Alpha
1.	0.9457
2.	0.9444
3.	0.9450
4.	0.9441
5.	0.9447
6.	0.9450
7.	0.9448
8.	0.9450
9.	0.9448
10.	0.9456
11.	0.9463
12.	0.9442
13.	0.9443
14.	0.9461
15.	0.9455
16.	0.9468
17.	0.9470
18.	0.9432
19.	0.9453
20.	0.9469
21.	0.9461
22.	0.9471
23.	0.9455
24.	0.9462
25.	0.9434
26.	0.9480
27.	0.9455
28.	0.9443
29.	0.9437
30.	0.9466
31.	0.9447
32.	0.9456
33.	0.9445
34.	0.9453
35.	0.9456
